# Prospective analysis of myocardial strain through the evolution of Chagas disease in the hamster animal model

**DOI:** 10.1007/s10554-021-02379-w

**Published:** 2021-09-18

**Authors:** Fernando Fonseca França Ribeiro, Henrique Turin Moreira, Antônio Carlos Leite de Barros-Filho, Denise M. Tanaka, Camila G. Fabricio, Luciano F. L. Oliveira, Cibele M. Prado, Marcus V. Simões, André Schmidt, Benedito C. Maciel, José A. Marin-Neto, Minna Moreira Dias Romano

**Affiliations:** 1 Cardiology Division, Internal Medicine Department, Cardiology Center of the Medical School of Ribeirão Preto, University of São Paulo Ribeirão Preto, Bandeirantes Avenue, 3900, Ribeirão Preto, São Paulo 14049-900 Brazil; 2 Physical Therapy Department, Federal University of Minas Gerais, Belo Horizonte, Brazil; 3 Animal Science and Food Engineering, University of São Paulo, USP, Pirassununga, São Paulo 13635-900 Brazil

**Keywords:** Echocardiography, Speckle-tracking, Strain, Chagas cardiomyopathy, Hamsters, Animal experimental model

## Abstract

Speckle tracking echocardiography (STE) enables early diagnosis of myocardial damage by evaluating myocardial strain. We aimed to study sequential changes in structural and ventricular functional parameters during Chagas disease (CD) natural history in an animal model. 37 Syrian hamsters were inoculated intraperitoneally with *Trypanosoma cruzi* (Chagas) and 20 with saline (Control). Echocardiography was performed before the infection (baseline), at 1 month (acute phase), 4, 6, and 8 months (chronic phase) using Vevo 2100 (Fujifilm Inc.) ultrasound system. Left ventricular end-diastolic diameter, Left ventricular end-systolic diameter (LVESD), Left ventricular ejection fraction (LVEF), Global longitudinal (GLS), circumferential (GCS) and radial (GRS) strain were evaluated. Tricuspid annular plane systolic excursion (TAPSE) was used to assess right ventricular function. At 8 months, animals were euthanized and LV myocardial samples were analyzed for quantitation of inflammation and fibrosis. LVEF decreased over time in Chagas group and a difference from Control was detected at 6 months (p-value of groups#time interaction = 0.005). There was a pronounced decrease in GLS, GCS and TAPSE in Chagas group (p-value of groups#time interaction = 0.003 for GLS, < 0.001 for GCS and < 0.009 for TAPSE vs Control) since the first month. LVESD, LVEF and GLS were significantly correlated to the number of inflammatory cells (r = 0.41, p = 0.046; r = − 0.42, p = 0.042; r = 0.41, p = 0.047) but not to fibrosis. In the Syrian hamster model of CD STE parameters (GLS and GCS) showed an early decrease. Changes in LVEF, LVESD, and GLS were correlated to myocardial inflammation but not to fibrosis.

## Introduction

Chagas disease (CD) remains one of the most prevalent infectious diseases in Latin America, where more than six million people are infected by the protozoan *Trypanosoma cruzi* [1, 2]. The World Health Organization (WHO) recognizes CD as one of the thirteen most neglected tropical diseases in the world, with 25 million people at risk of infection [3, 4]. Population interchange between endemic and non-endemic areas was responsible for the spread of the disease [5–7]. Consequently, CD has become an emerging public health issue also in non-endemic countries such as the United States and various European and Asian countries [8].

Chronic Chagas cardiomyopathy (CCC) is the most ominous manifestation of CD causing heart failure, conduction disturbances, ventricular arrhythmias, ventricular aneurysms, pulmonary and systemic thromboembolism, and sudden death [9, 10]. The pathogenesis of CCC is pleomorphic and involves various mechanisms related to parasite persistence causing low grade but incessant inflammation and also myocardial injury mediated by the immune system. A particular aspect of the pathogenesis of CCC is delayed myocardial damage, which is related to a persistently low intensity but incessant parasitism and myocardial injury mediated by the immune system [11]. Most patients require specialized care, anticoagulation, pacemaker, cardioverter-defibrillator implantation and heart transplantation, with a high overall medical and social burden [12, 13].

It is estimated that roughly 30 to 40% of individuals infected by the *T. cruzi* will develop CCC only 20 to 30 years after the initial infection [14]. This life-long characteristic of the natural history of CD, as also the absence of a prediction tool for who among infected people will develop CCC, is an unsolved challenge to clinical studies. In an attempt to overcome this problem, a model of *T. cruzi* infection in Syrian hamsters has been described to mimic all stages of CD with a more adequate timeline for research studies, aiming at the detection of early myocardial damage [15, 16].

Speckle tracking echocardiography (STE) has allowed the analysis of Left ventricle (LV) strain, a measurement of myocardial deformation, which has demonstrated higher accuracy for the detection of subclinical LV dysfunction in several clinical settings, such as cancer therapeutics-related cardiac dysfunction [17], idiopathic dilated cardiomyopathy [18–20], hypertrophic genetic cardiomyopathy [21], ischemic myocardial disease [22] and others [23]. The STE capability of diagnosing myocardial damage in the early stages of CD [24–26] and to predict evolution to CCC [27, 28] is still upon investigation.

The purpose of this study was to prospectively evaluate sequential changes in myocardial structure and function in an animal experimental model of CD using conventional and STE echocardiography compared to a control group. Specific objectives were (1) to examine the behavior of conventional echocardiographic systolic function parameters from both ventricles and LV myocardial deformation indices over lifelong evolution of animal experimental model of CD, compared to controls and (2) to evaluate the correlation between conventional echocardiography parameters and LV myocardial deformation indices with histopathologic changes.

## Methods

### Experimental animals

Twelve-week-old female hamsters (*Mesocricetus auratus*) were kept in a climatically controlled environment, with free access to water and standard chow and under a 12-h light/dark cycle. Animals were subjected to all experimental procedures under anesthesia with Ketamine (100 mg/Kg) and Xylazine (10 mg/Kg) to avoid pain and stress. Experimental protocols were approved by the Institutional Animal Research Ethics Committee (Protocol No. 013/2015-1). Exclusion criteria were: inability to sedate with a recommended dose of anesthetics; clinical evidence of extracardiac pathologies; the presence of cardiac arrhythmias such as bigeminism, ventricular and supraventricular tachycardia, and evidence of cardiac abnormalities such as congenital defects.

### Experimental protocol

Animals were inoculated intraperitoneally with 35,000 trypomastigote forms of *Trypanosoma cruzi* (Y strain) and had chronic infection confirmed by plasma antibodies (Chagas group) or injected with an equal volume of saline solution (Control group). Echocardiography was performed before the inoculation (baseline) and at disease timepoints of acute (1 month) and chronic (4, 6, and 8 months after) phases. Animals were observed through eight months and survivors were subjected to euthanasia and heart collection for histological analysis. To demonstrate significant differences in echocardiographic parameters, specially LVEF, with adequate statistical power, taking into account a predicted mortality of 50% in this experimental animal model [16], a minimum of 20 animals in each group would be necessary.

### Echocardiography

Echocardiogram was recorded using Vevo 2100 (Fujifilm Inc.) ultrasound system provided with a 30-MHz high-frequency transducer. Parasternal long-axis view (PSLAX) and short-axis view (PSSAX) were obtained. Left ventricular end-diastolic diameter (LVEDD), Left ventricular end-systolic diameter (LVESD), and Left ventricular ejection fraction (LVEF) were measured with M-mode at PSLAX. The LVEDD was measured in the maximum ventricular diastolic dimension and the LVESD at the maximum inward motion of the septum and posterior wall. LVEF was calculated by the method of Teichholz [29] (LVEF_Teichholz) and also by the bidimensional method of area-length (LVEF_2D). TAPSE was used to assess right ventricular function. All images were recorded by an echocardiographer experienced at imaging small animals at 5 to 10 min after sedation. All measurements represented the means of five consecutive cardiac cycles.

### Speckle tracking echocardiography

**Fig. 1:**
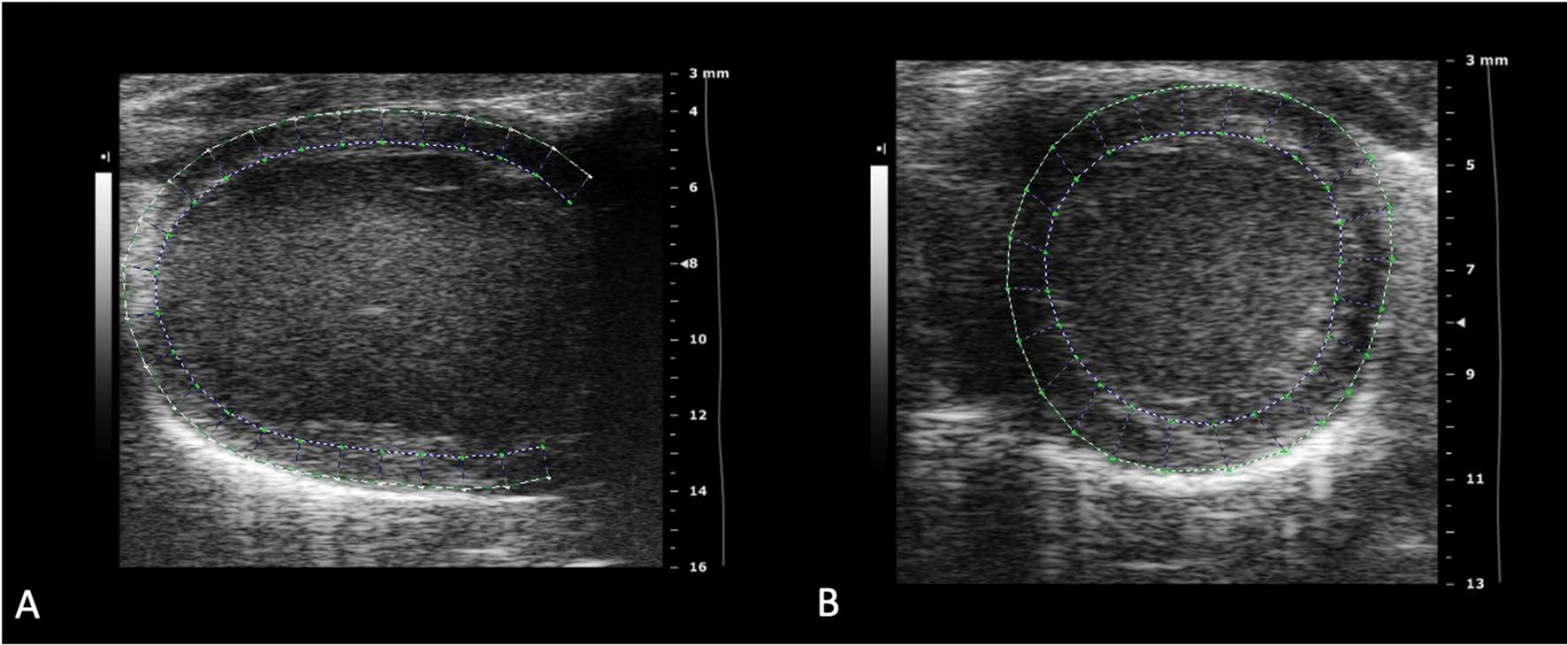
Myocardial strain analysis in hamsters from Parasternal long-axis view (PSLAX) and Parasternal short-axis view (PSSAX). LV strain was measured across a Region of interest (ROI) in the parasternal long-axis view (**A**) and short-axis view (**B**)

Left ventricular Global longitudinal strain (GLS) was obtained at PSLAX. LV Global circumferential strain (GCS) and LV Global radial strain (GRS) were evaluated at PSSAX at the papillary level. Two-dimensional speckle-tracking analysis was applied to assess LV deformation using the validated Vevo® Strain Software (VisualSonics Inc, Toronto, Canada) [30]. End-diastole was automatically defined at the peak of the QRS complex. End-systolic time was defined as the point of the smallest dimension of the LV cavity and visually checked with the use of mitral leaflets position at M-mode background image. Manual LV endocardial border delineation was performed in the PSLAX to measure GLS or PSSAX to measure GRS and GCS. Thereafter, epicardial borders were automatically defined, creating a Region of interest (ROI). If necessary, adjustments in ROI width were made to include the entire LV myocardium region, based on a six-segment model [31] in each projection (Fig. 1).

Finally, myocardium tracking was automatically obtained. Also, a visual tracking quality control was applied and a new tracing was performed when at least one segment was poorly tracked. In the presence of a persistent poor tracking of at least one segment per projection, global deformation analysis was considered not feasible. LV deformation was measured as the peak systolic change in the myocardial length relative to the length at end-diastole and represented by a percentage of change (%).

### Reproducibility

Reproducibility analysis for echocardiographic parameters of LV functional assessment was performed in 20 animals randomly selected. For intraobserver reproducibility, a rereading was done at least 90 days after the first reading, blinded to the previous results. For interobserver analysis, the second independent reader was blinded to the analysis of the first reader. Inter- and intrareader reproducibility analyses were evaluated using an Intraclass correlation coefficient (ICC) to assess absolute agreement and a Coefficient of variation (CV), determined by the standard deviation divided by the mean and expressed in percentage.

### Histopathological and morphometric analysis

Animals were killed, the hearts were rapidly removed, rinsed in 0.9% NaCl solution, and fixed in neutral buffered 10% formalin for histological study. For the histopathological study, the samples were dehydrated, clarified, embedded in paraffin, stained with hematoxylin and eosin and picrosirius red, and examined by light microscopy (n = 10–13/group). The tissue sections stained with hematoxylin and eosin were used to evaluate the intensity of inflammation. The number of inflammatory cells was determined by counting the number of mononuclear rounded interstitial cells (to exclude the spindle-shaped fibroblastic cells) in the myocardium of the left ventricles in 10 microscope fields (400 × magnification) per animal. The slides stained with picrosirius red were used to evaluate fibrosis through collagen quantification. To estimate the volume fraction (%) of fibrosis in picrosirius red-stained sections of the left ventricles, 10 microscope fields (400 × magnifications) were measured per animal.

For both morphometric analyses, the Leica QWin software (Leica Imaging Systems Ltd., Cambridge, England) in conjunction with a Leica microscope, video camera, and an online computer was used. Measurements were made by a skilled observed blinded to the groups.

### Statistical analysis

The normality of continuous data was assessed by histograms and the Shapiro–Wilk test. Continuous data are expressed as mean ± standard deviation (SD) if normally distributed, or as median [interquartile range] if not normally distributed. Categorical data are presented as absolute values and percentages. Wilcoxon–Mann–Whitney tests were used to evaluate the differences between the two groups at baseline examination. Correlation of fibrosis and inflammation with echocardiographic parameters of LV and RV structural and functional assessment was verified by Pearson’s correlation coefficient.

The analysis of variance for mixed models of repeated measures (mixed-ANOVA) was used to evaluate interaction (main effect) between the experimental groups (between-subject effect) and time (within-subject effect). Statistical analyses were performed using Stata 14.0 (StataCorp, College Station, TX) and P < 0.05 was considered statistically significant.

## Results

A total of 62 female Syrian hamsters was selected. Three animals were excluded from the basal evaluation, one of them because of thoracic deformity and two because of significant arrhythmias right after anesthesia. Two animals died during the first anesthesia. At baseline, 57 animals were subjected to echocardiography and there were no significant differences in characteristics between both groups, except for a higher weight of the Chagas group compared to controls (143 ± 12 g vs 130 ± 15 g; p = 0.004), Table 1. The overall mortality of all animals was 49%. The mortality of infected animals was 56.7%, with 27% occurring up to the end of the first month after infection. Table 2 shows the number of live animals over time.

**Table 1:** Baseline population characteristics

	Chagas	Control	p-value
n	37	20	
Age (days)	89 ± 1	89 ± 1	0.551
Weight (g)	143 ± 12	130 ± 15	0.004*
Heart rate (bpm)	198 ± 18	204 ± 18	0.277
LVEDD (mm)	6.6 ± 0.3	6.6 ± 0.3	0.575
LVESD (mm)	4.4 ± 0.4	4.3 ± 0.4	0.503
LVEF_Teichholz (%)	61 ± 5	64 ± 5	0.101
LVEF_2D (%)	57 ± 4	57 ± 4	0.961
TAPSE (mm)	1.5 ± 0.2	1.5 ± 0.2	0.81
GLS (%)	− 14.2 ± 3.4	− 15.2 ± 2.7	0.256
GCS (%)	− 20.4 ± 2.8	− 20.0 ± 2.2	0.55
GRS (%)	32.6 ± 9.5	33.7 ± 10.4	0.878

*LVEDD* left ventricle end diastolic dimension, *LVESD* left ventricle end systolic dimension, *LVEF_Teichholz* left ventricular ejection fraction calculated by Teichholz method, *LVEF_2D* two-dimensional left ventricular ejection fraction by area-length method, *TAPSE* tricuspid annular plane systolic excursion, *GLS* global longitudinal strain, *GCS* global circumferential strain, *GRS* global radial strain

*p < 0.05

**Table 2:** Number of live animals over time

	Baseline	1 Month	4 Months	6 Months	8 Months
Chagas (n)	37	27	20	19	16
Control (n)	20	18	15	14	13
Total (n)	57	45	35	33	29

**Fig. 2:**
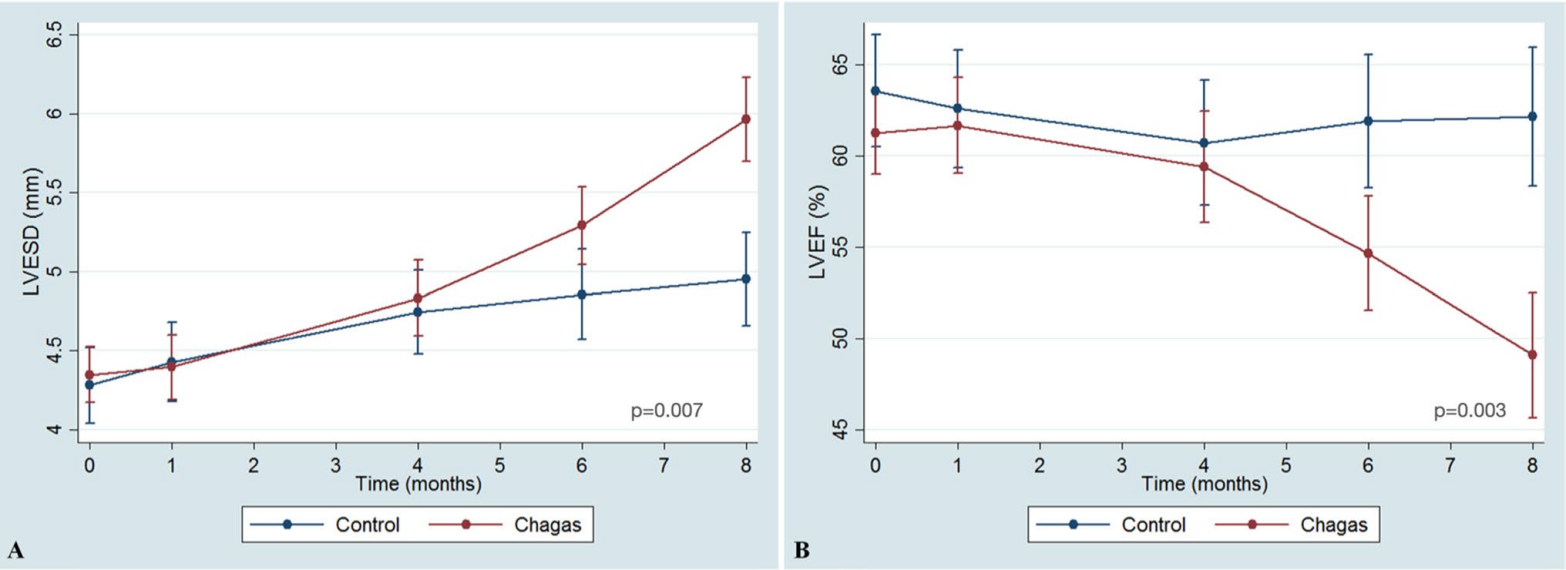
Left ventricle end-systolic dimension (LVESD) and ejection fraction (LVEF) through time in Chagas and Control groups. Panel **A** Behavior of LVESD over time in Chagas and Control groups. Panel **B** LVEF over time in Chagas and Control groups. *LVESD* left ventricular end-systolic diameter, *LVEF_2D* two-dimensional derived left ventricular ejection fraction by the area-length method. Comparison between groups through time with mixed model ANOVA

During the follow-up time, the sequential analysis showed that Chagas group had a significant increase in the LVESD and a decrease in LVEF_2D in comparison with Control group (p-value of the interaction groups#time = 0.007 for LVESD and of 0.003 for LVEF_2D) Fig. 2, Panels A and B.

**Fig. 3:**
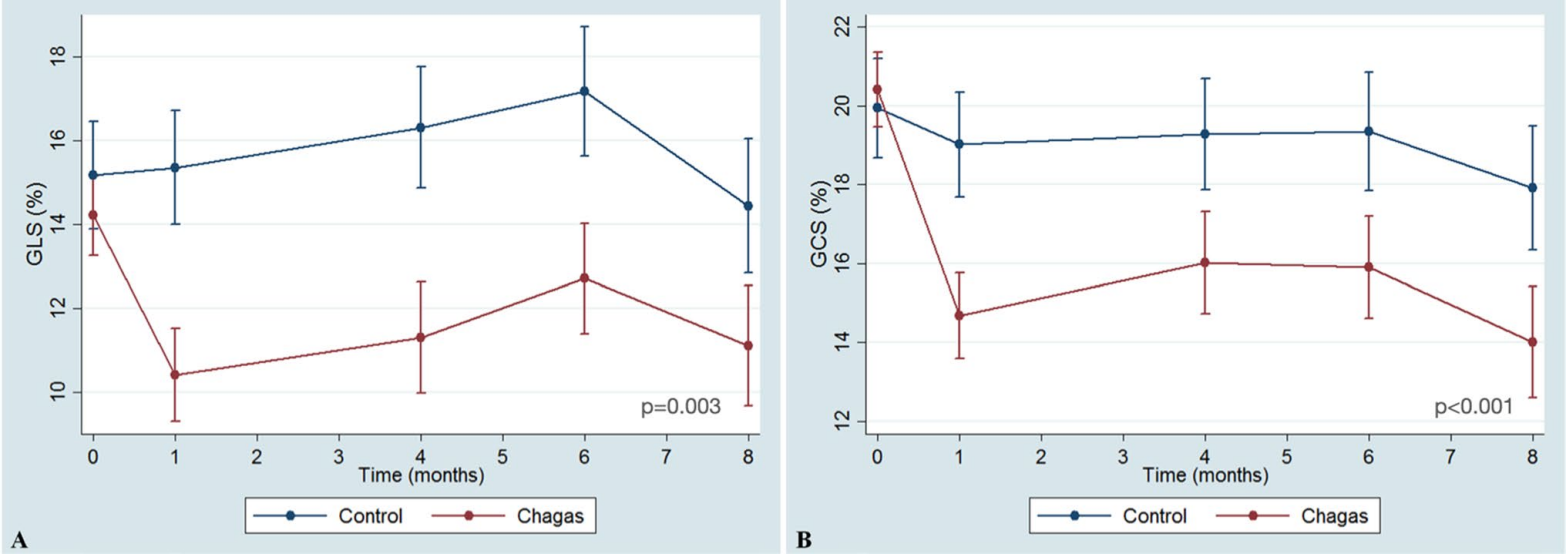
Deformation echocardiographic parameters evolution over time in Chagas and Control groups. Panel **A** Evolution of GLS over time in Chagas and Control groups. Panel **B** Evolution of GCS over time in Chagas and Control groups. *GLS* global longitudinal strain, *GCS*: global circumferential strain. Comparison between groups through time with mixed model ANOVA

Additionally, GLS and GCS of Chagas group showed a significant reduction over time in Chagas group compared to the Control group (p-value of the interaction groups#time = 0.003 for GLS and < 0.001 for GCS) - Fig. 3, Panels A and B. For both parameters of myocardial deformation, the difference between the two groups is verified from the first month of evaluation.

The evolution of both ventricles’ structure and functional parameters is presented in Table 3 for Control group and in Table 4 for Chagas group. Values of LVEF_2D in Chagas group were 57 ± 4% at baseline and reduced to 42 ± 13% at 8 months. GLS in Chagas group was − 14.2 ± 3.4% at baseline and reduced to − 10.4 ± 3.0% from the first month.

**Table 3:** Sequential assessment of biventricular structure and function in the control group

	Baseline	1 Month	4 Months	6 Months	8 Months
LVEDD (mm)	6.6 ± 0.3	6.8 ± 0.3	7.2 ± 0.3	7.4 ± 0.3	7.6 ± 0.5
LVESD (mm)	4.3 ± 0.4	4.4 ± 0.3	4.7 ± 0.3	4.8 ± 0.4	4.9 ± 0.5
LVEF_Teichholz (%)	63 ± 5	63 ± 3	61 ± 4	62 ± 5	62 ± 5
LVEF_2D (%)	57 ± 4	57 ± 3	55 ± 3	57 ± 6	53 ± 3
GLS (%)	− 15.2 ± 2.6	− 15.4 ± 2.4	− 16.3 ± 2.9	− 19.3 ± 2.4	− 15.0 ± 2.0
GCS (%)	− 20.0 ± 2.2	− 19.0 ± 2.1	− 19.0 ± 2.0	− 16.0 ± 2.6	− 18.0 ± 3.0
GRS (%)	34.0 ± 10.0	31.0 ± 4.5	29.0 ± 6.0	30.0 ± 5.6	29.0 ± 9.3
TAPSE (mm)	1.5 ± 0.2	1.7 ± 0.2	1.6 ± 0.2	1.7 ± 0.1	1.7 ± 0.2

The analysis of variance (mixed-ANOVA) for mixed models of repeated measures was used to evaluate the differences between the two groups over time

*LVEDD* left ventricle end diastolic dimension, *LVESD* left ventricle end systolic dimension, *LVEF_Teichholz* left ventricular ejection fraction calculated by Teichholz method, *LVEF_2D* two-dimensional left ventricular ejection fraction by area-length method, *GLS* global longitudinal strain, *GCS* global circumferential strain, *GRS* global radial strain, *TAPSE* tricuspid annular plane systolic excursion

**Table 4:** Sequential assessment of ventricular structure and function in the Chagas group

	Baseline	1 Month	4 Months	6 Months	8 Months
LVEDD (mm)	6.6 ± 0.3	6.7 ± 0.6	7.2 ± 0.4	7.5 ± 0.5	8.0 ± 0.6
LVESD (mm)	4.4 ± 0.4	4.4 ± 0.5	4.8 ± 0.5	5.3 ± 0.7	6.0 ± 1.2
LVEF_Teichholz (%)	61 ± 5	62 ± 7	59 ± 6	55 ± 9	49 ± 14
LVEF_2D (%)	57 ± 4	55 ± 6	56 ± 6	48 ± 8	42 ± 13
GLS (%)	− 14.2 ± 3.4	− 10.4 ± 3.0	− 11.3 ± 2.4	− 12.7 ± 2.8	− 11.1 ± 3.5
GCS (%)	− 20.6 ± 2.6	− 14.7 ± 3.2	− 16.0 ± 3.0	− 15.9 ± 2.6	− 14.0 ± 4.3
GRS (%)	32.6 ± 9.5	24.6 ± 7.5	23.8 ± 6.5	24.0 ± 6.9	22.7 ± 8.8
TAPSE (mm)	1.5 ± 0.2	1.3 ± 0.2	1.4 ± 0.2	1.4 ± 0.2	1.4 ± 0.2

The analysis of variance (mixed-ANOVA) for mixed models of repeated measures was used to evaluate the differences between the two groups over time

*LVEDD* left ventricle end diastolic dimension, *LVESD* left ventricle end systolic dimension, *LVEF_Teichholz* left ventricular ejection fraction calculated by Teichholz method, *LVEF_2D* two-dimensional left ventricular ejection fraction by area-length method, *GLS* global longitudinal strain, *GCS* global circumferential strain, *GRS* global radial strain, *TAPSE* tricuspid annular plane systolic excursion

**Fig. 4:**
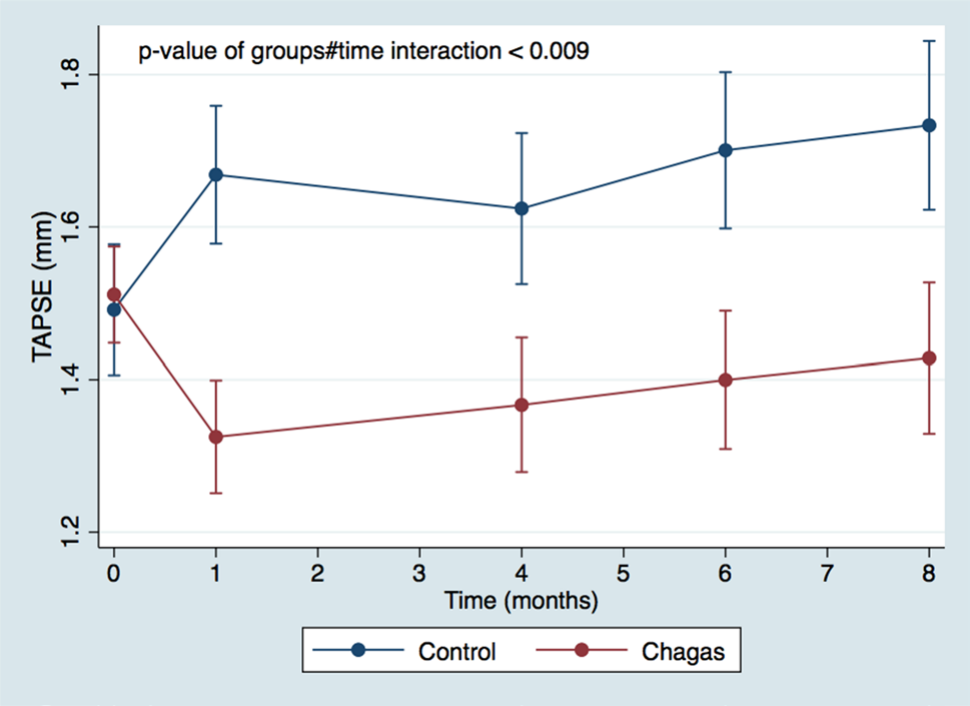
Evolution of TAPSE over time in Chagas and Control groups. *TAPSE* tricuspid annular plane systolic excursion. Comparison between groups through time with mixed model ANOVA

The TAPSE index of the Chagas group also presented a significant reduction over time compared to the Control group (p-value of interaction groups#time < 0.009) - Fig. 4, with the difference also observed since the first month post-infection.

### Histological analysis

**Fig. 5:**
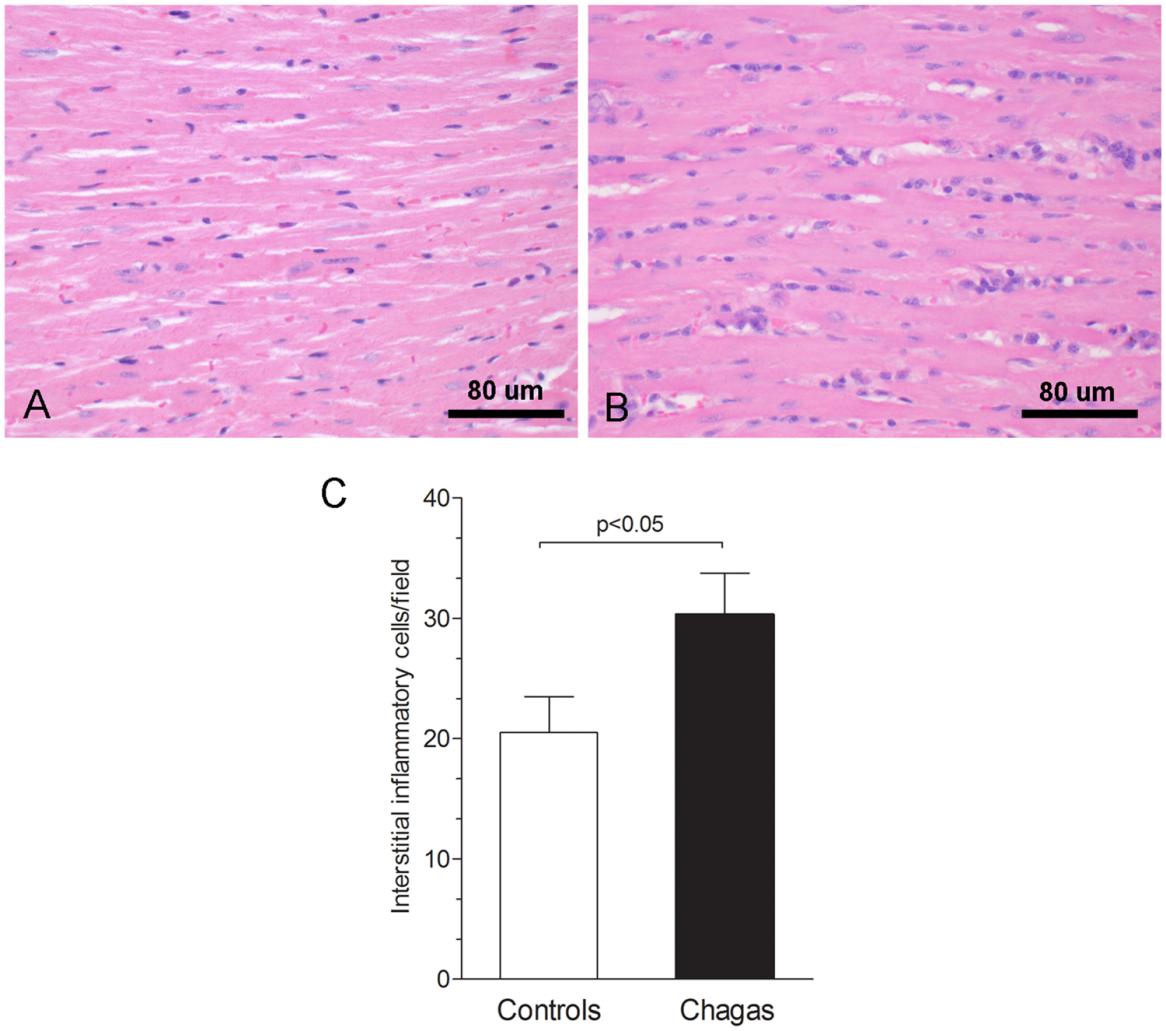
Quantitative histological analysis of myocardial inflammation in Control group (**A**) and Chagas group (**B**). Graph representing the greater number of inflammatory cells in animals with Chagas disease compared to animals in the control group (**C**)

Histological analysis demonstrated diffuse myocarditis characterized by lymphomononuclear interstitial infiltrate in the Chagas group. There was an increase of 50% in the amount of mononuclear rounded interstitial cells (30.30 ± 10.9 vs 20.5 ± 9.46; p = 0.045) (Fig. 5).

**Fig. 6:**
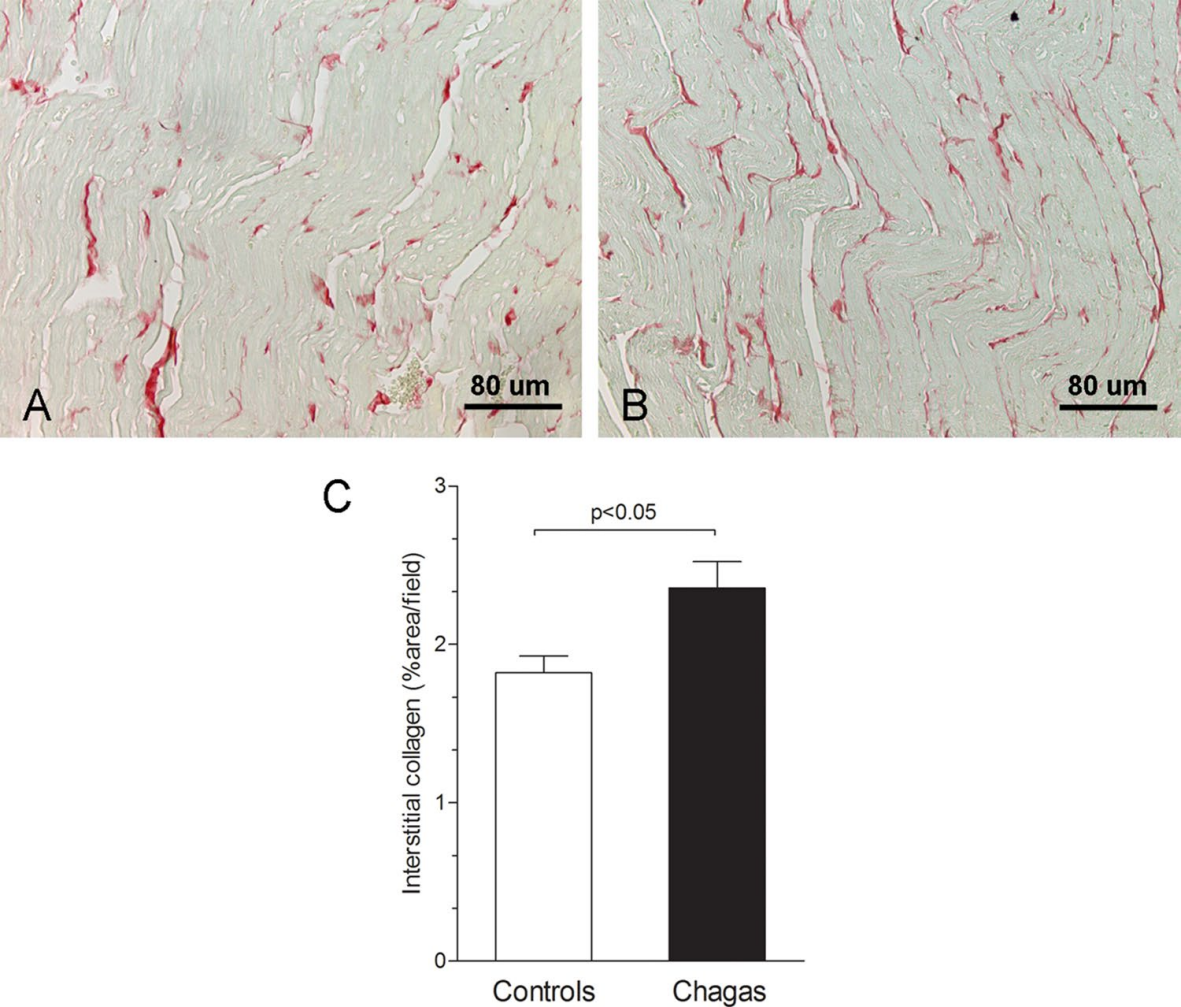
Quantitative histological analysis of interstitial myocardial fibrosis in Control group (**A**) and Chagas group (**B**). Graphic representing the highest percentage of interstitial fibrosis in animals with Chagas disease compared to animals in the control group (**C**)

The analysis of picrosirius red-stained sections revealed mild myocardial fibrosis manifested by an increased amount of pericellular collagen (endomysial matrix). The increase in the volume fraction of fibrosis was around 30% (2.36 ± 0.58 vs 1.82 ± 0.29; p = 0.027) (Fig. 6) in the Chagas group compared to the Control group.

There was a significant moderate correlation between inflammation and GLS (r = 0.41, p = 0.047), as also to LVEF_Teichholz (r =− 0.42, p = 0.042) and LVESD (r = 0.041, p = 0.046), Table 5. There was no significant correlation between systolic LV functional parameters and myocardial fibrosis.

**Table 5:** Correlation of echocardiographic parameters with histological analysis

	Inflammation		Fibrosis	
	Pearson’s r	p-value	Pearson’s r	p-value
LVEDD	0.28	0.19	0.15	0.50
LVESD	0.41	0.046*	0.18	0.42
LVEF_Teichholz	− 0.42	0.042*	− 0.21	0.33
LVEF_2D	− 0.24	0.25	− 0.13	0.54
GLS	0.41	0.047*	− 0.35	0.10
GCS	0.12	0.57	− 0.33	0.13
GRS	− 0.22	0.30	− 0.19	0.38
TAPSE	− 0.39	0.07	− 0.36	0.10

*LVEDD* left ventricle end diastolic dimension, *LVESD* left ventricle end systolic dimension, *LVEF_Teichholz* left ventricular ejection fraction calculated by Teichholz method, *LVEF_2D* two-dimensional left ventricular ejection fraction by area-length method, *GLS* global longitudinal strain, *GCS* global circumferential strain, *GRS* global radial strain, *TAPSE* tricuspid annular plane systolic excursion

*p < 0.05

### Reproducibility

All echocardiographic parameters for LV functional assessment revealed good interobserver reproducibility, with the intraclass correlation coefficient ranging from 0.52 (for LVEF_2D) to 0.91 (for GLS). LVEF by Teichholz’s method showed the lowest interobserver coefficient of variation (5.66%), while GRS exhibited the highest (13.63%).

Intraobserver reproducibility showed an intraclass correlation coefficient ranging from 0.49 (for LVEF_2D) to 0.92 (for GLS) and a coefficient of variation from 4.25% (for LVEF by Teichholz’s method) to 10.85% (for GRS) (Table 6).

**Table 6:** Reproducibility of echocardiographic parameters to assess LV systolic function

	Interobserver analysis		Intraobserver analysis	
	ICC	CV (%)	ICC	CV (%)
LVEF (Teichholz)	0.86	5.66	0.91	4.25
LVEF_2D	0.52	7.24	0.49	9.26
LVEF (*st*)	0.74	8.37	0.90	5.53
GLS	0.91	11.10	0.92	7.99
GCS	0.77	7.41	0.85	7.10
GRS	0.67	13.63	0.73	10.85

*CV* coefficient of variation, *ICC* intraclass correlation coefficient, *LVEF* (*Teichholz*) left ventricular ejection fraction by Teichholz method, *LVEF_2D* two-dimensional left ventricular ejection fraction by area-length method, *LVEF* (*st*) left ventricular ejection fraction by speckle tracking method, *GLS* global longitudinal strain, *GCS* global circumferential strain, *GRS* global radial strain

## Discussion

The present study evaluated biventricular geometrical and functional ventricular changes in an experimental model of Chagas disease in Syrian hamsters over time in comparison with a Control group.

The findings revealed that parameters of longitudinal and circumferential myocardial deformation obtained by STE are significantly depressed early after the infection with *T. cruzi*. In contrast, LVESD and LVEF showed changes at an advanced time point of CD, when mortality is high. Besides, the present investigation indicates an early involvement of the right ventricle in animals infected with *T. cruzi*, before the overt dysfunction of LV.

### Evolution of geometrical and biventricular functional parameters in the experimental animal model of CD in hamsters

Although the phases and forms of CD are difficult to be reproduced in rodent models of *T. cruzi* infection, in Syrian hamsters CD can be replicated through all clinical stages including the acute phase, the latent period (indeterminate), the chronic cardiomyopathy, with myocardial dysfunction caused by multifocal and diffuse myocarditis and interstitial fibrosis. The first month after infection in hamsters presents the acute phase of the disease, with a high mortality rate. In our study, we observed 27% of mortality at this point and this finding is similar to that reported in a previous study, which showed a mortality rate of 33% in the first month [16]. After the acute phase, survivors evolve to an apparent quiescent period, with no clinical manifestations neither evidence of overt myocardial systolic dysfunction, similarly to the indeterminate form [32] of chronic CD in humans. Prior studies using the same animal experimental model did not find LV myocardial impairment with conventional echocardiography until 6 months after the initial infection [33]. In our study, LVEF and LVESD were reduced in infected animals compared to controls at 6 months of disease evolution, suggesting that the latent phase of CD in this experimental animal model may be shorter than previously reported [15, 16, 34]. It is also plausible to assume that those differences could be explained, at least in part, by the different echocardiographic techniques applied [15], which, in our study, used a higher spatial resolution ultrasound system.

Other studies reported on late LV dilatation in hamsters infected with *T. cruzi* [15]. In our study, LVEDD was not different in infected animals from controls until the end of experimentation time at 8 months. This finding was also observed in the investigation from Tanaka et al. and may suggest not only LVEDD is a very late marker of CCC but also it could not represent LV dysfunction [33]. Our study was the first to assess two-dimensional derived LVEF by the area-length method in *T. cruzi* infected Syrian hamsters. Previous studies had already shown the feasibility of two-dimensional LVEF in mice and rat models of surgical myocardial overload, infarction, and LV dysfunction [35]. However, as CCC is typically a segmental disease [36], M-mode derived LVEF (Teichholz method) may not accurately estimate LV systolic dysfunction.

This was also the first study to prospectively evaluate RV function through the evolution of CD in Syrian hamsters. Our data showed RV is early compromised. These findings corroborate the results from studies in humans that have shown early changes of RV geometry and function even in the indeterminate form of CD [37–39]. The damage to the RV imposed by CD was previously demonstrated in studies of RV myocardial tissue samples [40] and in a murine model of disease [41] but only in our investigation a sequential evolution of RV dysfunction has been described over time.

### Speckle tracking echocardiography as a tool for early detection of myocardial dysfunction in an animal experimental model of CD

This was the first study to use STE in Syrian hamsters infected with *T. cruzi*. Our results demonstrated that GLS and GCS were able to detect early myocardial damage in animals infected with the protozoan when compared with control animals at the same age. It is noteworthy that the change in these parameters occurred despite normal values of LVEF, as early as one month after infection, a moment recognized in this model as the acute phase of the CD when mortality rates are high.

There have been some studies with STE in patients with Chagas disease. However, the behavior of myocardial deformation through all stages of the disease in a sequential analysis had not been previously demonstrated. Although in CCC global LV deformation parameters and LVEF are reduced [25, 42], results are still conflicting about the early stages of the disease [24, 26, 43]. Few clinical studies evaluated LV myocardial deformation in patients with the indeterminate form of CD. One study showed that LV radial strain was reduced in 32 patients compared to controls, but statistical significance was achieved only at trend analysis [25]. Gomes et al. did not encounter STE differences between controls and patients at the early stages of the disease, except for a small group of seven patients who presented significant fibrosis diagnosed by cardiac magnetic resonance [24].

In contrast to the reduction of LV GLS and GCS observed in infected animals since the very early phase of the disease, GRS was not different between Chagas and control groups in our study. Radial strain interpretation has been challenging in several aspects. The wide variability of GRS indexes and also low reproducibility are recognized even in normal human hearts [44]. Also, when providing normal reference intervals of STE for the Syrian hamster model, GRS presented the lowest values of reproducibility [45]. Technically the lower reproducibility of GRS is comprehensible in any, human or small animal experimental images [46], based on the difficulty not to measure strain, but to track speckles in lateral lobes of bidimensional LV images. Measurements of radial strain are made from parasternal short-axis views, where segments in lateral lobes of the ultrasound beam do not have enough spatial resolution to provide adequate tracking [47], Additionally, radial deformation may be enhanced at the initial phases of myocardial damage, as a compensatory mechanism to the reduction of longitudinal and circumferential deformation [23, 48]. Thus, clinical use and interpretation of radial strain should be cautious. Lima et al., in concordance with our findings, also did not show differences in radial strain in Chagas patients at the early stages of the disease [49].

Measurements of myocardial deformation in this study strictly followed the latest published guidelines [48]. EKG signals were always recorded, end-systolic time was marked and strain peak was always measured as a systolic peak [47]. Definitions about what strain peak was measured were not always clarified in previous studies using STE in CD [25, 26]. Normal values of deformation parameters of LV in Syrian hamsters were previously published by our research group [50]. In pathologies such as CCC, several LV conduction disturbances may be present and hence some LV segments can show post systolic peak deformations, which may not accurately represent patients’ myocardial systolic function [23].

### Histological analysis

Corroborating previous findings from our laboratory [16, 51] histologic data presented in this study show an increase of both, myocardial inflammatory cells and interstitial fibrosis in *T. cruzi* infected animals compared to controls. The low extent of myocardial fibrosis (4%) corroborates this model as a representative of initial myocardial damage when studied until 8 months. Previous studies show fibrosis of 10% of the whole LV myocardial area when animals reach 10 months of infection [16]. Despite the lower incidence of fibrosis, GLS correlation to inflammatory cell count reaffirms this deformation parameter as an early marker of myocardial damage in Chagas disease’s physiopathology, even before significant fibrosis. Other mechanisms which could be present in Chagas cellular damage as myocardial disarray [52, 53] were not explored in this study.

## Limitations

Our findings should be interpreted in the context of the various limitations. First, the fact that only animals who had all echocardiographic analyses in pre-specified time points were included, according to the inherent characteristic of mixed-ANOVA analysis [54]. Although our experimental study design had the power to demonstrate significant differences between myocardial structural and functional echocardiographic parameters over time, there is a natural selection bias, because animals that spontaneously died before 8 months could not be included in the mixed-ANOVA analysis. Second, it is important to consider that anesthetic agents could influence clinical parameters such as heart rate, blood pressure, and, in the last instance, myocardial performance. Although isoflurane is considered by some authors as a more superficial anesthetic agent, it has significant effects on cardiac function evaluated by LVEF [55], ketamine has shown fewer effects on LVEF, and its results were already tested in hamsters [56]. There is a lack of data in the literature about the impact of anesthetic agents in deformation myocardial parameters, especially in this Syrian hamster experimental model. Nonetheless, there were no differences in cardiac HR between the two groups over time in our present investigation. Third, there was no LV diastolic analysis based on difficulties to acquire apical long-axis views of this animal model. Finally, there was no RV tissue analysis to correlate to functional echocardiographic parameters.

## Translational aspects and future perspectives

Results of this investigation in the animal model of *T. cruzi* infection suggest STE as a potentially useful tool to study early sequential myocardial damage through CD evolution in humans. GRS and GLS can show myocardial impairment before the overt LV dysfunction shown by a reduction in LVEF. There is no robust evidence about how frequently echocardiographic examinations should be done in serological-positive patients without clinical signs of disease [57]. However, our experimental data suggest sequential STE evaluations could show early reductions in LV deformation parameters. STE could be useful in selecting patients that should be monitored closely and, eventually, select patients who could benefit from early treatment. Also, early detection of impairment in RV functional parameters may be a marker of disease progression from the indeterminate to the CCC form of CD.

Future studies delineated to investigate the prognostic value of STE in CD are warranted. Syrian hamster experimental animal model could provide insights to therapeutic effects of drugs in *T. cruzi* infection before LVEF reductions and testing reversibility of deformation parameters and myocardial function improvement.

## Conclusions

In the Syrian hamster experimental model of CD, the left ventricular STE parameters (GLS and GCS) showed an early decrease, and RV dysfunction was also detected even before overt LV dysfunction. Changes in LVEF, LVESD, and GLS were correlated to myocardial inflammation but not to fibrosis.

## Abbreviations

CCC: Chronic Chagas cardiomyopathy
STE: Speckle tracking echocardiography
LV: Left ventricle
CD: Chagas disease
PSLAX: Parasternal long-axis view
PSSAX: Parasternal short-axis view
LVEDD: Left ventricular end-diastolic diameter
LVESD: Left ventricular end-systolic diameter
LVEF: Left ventricular ejection fraction
LVEF_Teichholz: LVEF calculated by applying the method of Teichholz
LVEF_2D: LVEF calculated by bidimensional method of area-length
TAPSE: Tricuspid annular plane systolic excursion
GLS: Global longitudinal strain
GCS: Global circumferential strain
GRS: Global radial strain
ROI: Region of interest
RV: Right ventricle
